# Using an incentive spirometer reduces pulmonary complications in patients with traumatic rib fractures: a randomized controlled trial

**DOI:** 10.1186/s13063-019-3943-x

**Published:** 2019-12-30

**Authors:** Shao-Kai Sum, Ya-Chuan Peng, Shun-Ying Yin, Pin-Fu Huang, Yao-Chang Wang, Tzu-Ping Chen, Heng-Hsin Tung, Chi-Hsiao Yeh

**Affiliations:** 1 Department of Thoracic and Cardiovascular Surgery, Chang Gung Memorial Hospital, 222 Mai-Chin Road, Keelung, 204 Taiwan, Republic of China; 2 Department of Nursing, Chang Gung Memorial Hospital, Keelung, Taiwan; 3 School of Nursing, National Taipei University of Nursing and Health Science, Taipei, Taiwan; 4 School of Medicine, Chang Gung University, Tao-Yuan, Taiwan

**Keywords:** Incentive spirometer, Rib fracture, Pulmonary complications, Forced vital capacity, Forced expiratory volume

## Abstract

**Background:**

An incentive spirometer (IS) is a mechanical device that promotes lung expansion. It is commonly used to prevent postoperative lung atelectasis and decrease pulmonary complications after cardiac, lung, or abdominal surgery. This study explored its effect on lung function and pulmonary complication rates in patients with rib fractures.

**Methods:**

Between June 2014 and May 2017, 50 adult patients with traumatic rib fractures were prospectively investigated. Patients who were unconscious, had a history of chronic obstructive pulmonary disease or asthma, or an Injury Severity Score (ISS) ≥ 16 were excluded. Patients were randomly divided into a study group (*n* = 24), who underwent IS therapy, and a control group (*n* = 26). All patients received the same analgesic protocol. Chest X-rays and pulmonary function tests (PFTs) were performed on the 5th and 7th days after trauma.

**Results:**

The groups were considered demographically homogeneous. The mean age was 55.2 years and 68% were male. Mean pretreatment ISSs and mean number of ribs fractured were not significantly different (8.23 vs. 8.08 and 4 vs. 4, respectively). Of 50 patients, 28 (56%) developed pulmonary complications, which were more prevalent in the control group (80.7% vs. 29.2%; *p* = 0.001). Altogether, 25 patients had delayed hemothorax, which was more prevalent in the control group (69.2% vs. 29.2%; *p* = 0.005). Two patients in the control group developed atelectasis, one patient developed pneumothorax, and five patients required thoracostomy. PFT results showed decreased forced vital capacity (FVC) and forced expiratory volume in 1 s (FEV_1_) in the control group. Comparing pre- and posttreatment FVC and FEV_1_, the study group had significantly greater improvements (*p* < 0.001).

**Conclusions:**

In conclusion, the use of an IS reduced pulmonary complications and improved PFT results in patients with rib fractures. The IS is a cost-effective device for patients with rib fractures and its use has clinical benefits without harmful effects.

**Trial registration:**

ClinicalTrials.gov, NCT04006587. Registered on 3 July 2019.

## Introduction

Chest trauma patients account for around 10–15% of all traffic accident victims. In the United States, 400,000 patients are admitted to hospital annually due to chest trauma [1]. Chest trauma is the second highest cause of mortality due to traffic accidents, accounting for about 25% of deaths, which is only slightly lower than for deaths due to head injuries [2]. Chest trauma may involve a variety of organs, including the heart, great vessels, lungs, trachea, and the esophageal or chest wall. Rib fracture is one of the most common injuries, accounting for about 61–90% of injuries [3]. Multiple complications can follow rib fracture, including pneumothorax, hemothorax, lung contusion, flail chest, atelectasis, respiratory failure, and even death. Atelectasis is the most common complication [2–8]. The mortality rate related to rib fracture has been reported to be 10–12% [9, 10] and is dependent on the severity of the intra- and extra- thoracic injuries, age, heart disease, diabetes mellitus, pneumonia, and the number of fractured ribs [11–14]. Rib fracture patients commonly complain about chest pain, which is caused by damaged lung hygiene, obstruction of the lower airway, and subsequent atelectasis and hypoventilation. Hypoxemia, pneumonia, respiratory failure, and other morbidities may cause lengthy hospital stays and mortality [2–4, 6, 15]. Rib fracture patients do not usually require surgical intervention. However, admission for pain control and further observation are required to maintain lung hygiene and prevent further complications.

An incentive spirometer (IS) is a mechanical device that helps lung expansion. It is commonly used to prevent postoperative lung atelectasis and decreased pulmonary complications after cardiac, lung, or abdominal surgery. It can increase the maximal inspiration capacity and lung compliance, improve oxygenation, and maintain the patency of lower airways to prevent and treat atelectasis [12, 16–22]. This study explored the effects of using an IS on lung function and pulmonary complication rate in rib fracture patients.

## Methods

After institutional review board approval (Chang Gung Medical Foundation, reference 103-0421A3; ClinicalTrials.gov Identifier: NCT04006587), we recruited adult patients (>18 years old) seen at our institution between June 2014 until May 2017 with traumatic rib fractures. Written inform consent was obtained from all participants. For inclusion, a patient had to have at least one rib fracture as detected by a chest X-ray or computed tomography scan.

**Fig. 1:**
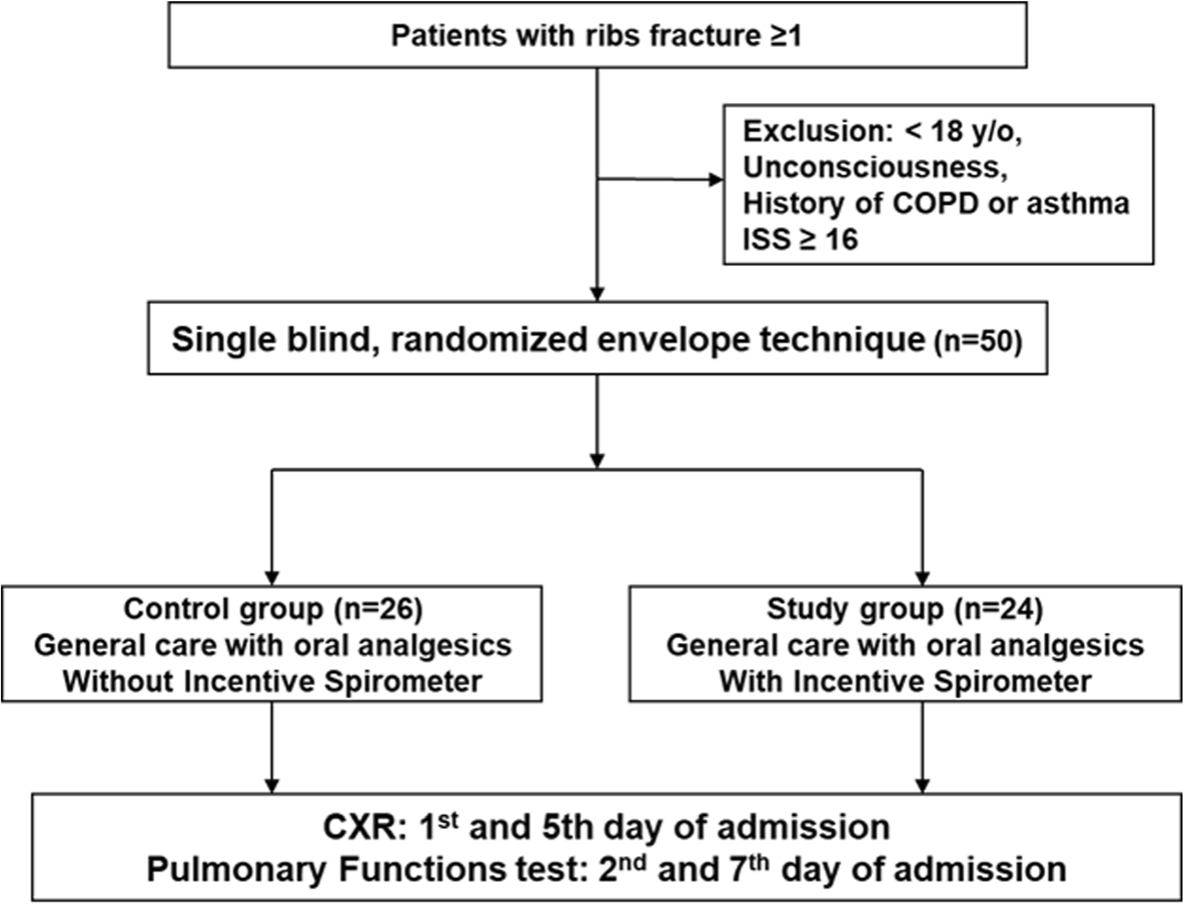
Study flow chart. COPD chronic obstructive pulmonary disease, CXR chest X-ray, ISS Injury Severity Score, y/o years old

A total of 50 patients were enrolled into this study. We excluded patients who were unconscious, had a history of chronic obstructive pulmonary disease or asthma, or an Injury Severity Score (ISS) ≥ 16. Patients were randomly allocated into two groups using sealed envelopes [23]: a study group (*n* = 24) who were advised to use the IS and a control group (*n* = 26) who did not use the IS (Fig. 1). A flow-oriented Tri-flow® (model TB-93100; Besmed Health Business Corporation, New Taipei City, Taiwan) was used. These devices are easy to use and the results are clearly visible, with the three floating balls indicating inspiratory flows of 600, 900, and 1200 ml/s. All patients were managed with the same oral analgesic protocol. A chest X-ray was obtained on the 1st and 5th days of admission by a radiologist. A pulmonary function test (PFT; Microspiro HI298, Chest Corporation, Tokyo, Japan) was performed on the 2nd and 7th days of hospitalization by a pulmonologist. The results were defined as the pre-test and post-test, respectively. Each patient’s forced vital capacity (FVC) and forced expiratory volume in 1 s (FEV_1_) were recorded. The predictive values (%FVC predictive and %FEV_1_ predictive) for each patient were individualized according to weight, height, sex, and age. According to Ruppel [24] normal values are FVC > 80%, FEV_1_ > 75%, FEV_1_ / FVC (%) > 75%, and flow rate > 60%. A numeric rating scale (NRS) was used to define the severity of chest pain on the 1st and 5th days of admission.

In the study group, patients were shown how to use the IS in a seated or semi-seated position. They were instructed to maintain a sustained maximal inspiration for 3–5 s before exhalation, ten times per hour, for at least 8 hours a day [25]. The primary outcome for this group was pulmonary complication rate, including complications such as atelectasis, pneumonia, hemothorax, and pneumothorax. The secondary outcomes were lung function test measurements (including %FVC and %FEV_1_), length of hospital stay, and chest pain as assessed by the NRS. The discharge criteria were stable vital signs, absence of pulmonary complications (such as atelectasis, pneumonia, pneumothorax, and hemothorax), and acceptable pain (NRS < 4).

### Statistical analysis

Categorical variables, such as sex, smoking status, rib fracture site, number of ribs fractured, trauma mechanism, associated injuries, comorbidities, and use of anti-coagulants or anti-platelets, were summarized with counts and percentages. Continuous variables, such as age, height, weight, body mass index (BMI), and ISS were summarized with means and standard deviation. An analysis of covariance (ANCOVA) was used to evaluate pre- and post-test lung function in the two groups. Between-group comparisons of the outcomes after IS use, including pulmonary complications, PFT results, length of hospitalization, and pain scores, were analyzed using an independent *t*-test, a chi-squared test, and a paired *t*-test. All *p* values were two-sided, and *p* < 0.05 was considered statistically significant. The sample size was determined with G*Power (version 3.1.9.4, Universität Kiel, Germany), which found that 25 subjects per group were needed to provide 80% power to detect the results as significant at α = 5% [26]. All statistical analyses were performed using SPSS version 20 (IBM, Armonk, NY, USA).

## Results

### Demographic information

Between June 2014 and May 2017, 50 patients with traumatic rib fractures who were admitted to our institution were recruited into this study after providing written inform consent. The patients were prospectively and randomly divided into two groups using sealed envelopes. There were no differences in age or gender between the groups. Patients in the study group (*n* = 24) were advised to use the IS and the other group was not (control group; *n* = 26). Their average age was 55.2 ± 14.5 years (range 18 to 88 years) and 34 patients (68%) were male. The mean number of ribs fractured was 3.94 ± 2.05 (study group 3.79 ± 2.21; control group 4.08 ± 1.94; *p* = 0.341). The number of patients with three or more fractured ribs was 39 (78%) (control group 21 [80.8%]; study group 18 [75%]; not significant). The mean ISS was 8.16 ± 3.76 (study group 8.08 ± 3.78; control group 8.23 ± 3.81; *p* = 0.894). The site of rib fracture did not differ between the groups, and only four patients (8%) had bilateral rib fractures, one in the study group and three in the control group. The most common mechanism of trauma was a motorcycle accident (about 50%), and the second most common was a fall from at least 3 m (about 24%). Associated injuries included eight patients with clavicle fractures, five patients with scapular fractures, four patients with bone fractures of the extremities, one patient with head injuries, and one patient with a liver laceration. Only five patients were using anti-platelets or anti-coagulants before the trauma, three in the study group and two in the control group. There were no statistically significant differences in smoking history, comorbidities, trauma mechanism, or BMI between the two groups (Table 1).

**Table 1:** Patient demographics

	Whole cohort *n* = 50 (%)	Control group *n* = 26 (%)	Study group *n* = 24 (%)	*p* value
Age (years)	55.2 ± 14.5	54.5 ± 15.2	56.0 ± 13.9	0.904
Sex (male, %)	34 (68%)	17 (65.4%)	17 (70.8%)	0.680
Height (cm)	164.0 ± 7.9	163.0 ± 7.4	165.0 ± 8.5	0.216
Weight (kg)	69.0 ± 12.0	70.9 ± 10.9	66.8 ± 12.9	0.281
BMI (kg/m^2^)	25.6 ± 4.2	26.7 ± 4.7	24.4 ± 3.2	0.493
ISS	8.2 ± 3.8	8.2 ± 3.8	8.1 ± 3.8	0.894
Initial blood laboratory data				
White blood cell (10^3^/uL)		15.3 ± 5.4	12.3 ± 5.0	0.466
Hemoglobin (g/dL)		12.6 ± 2.9	13.7 ± 1.7	0.500
Hematocrit (%)		37.1 ± 7.5	39.9 ± 4.3	0.466
Platelet count (10^3^/uL)		225.4 ± 63.6	209.7 ± 44.2	0.518
Prothrombin time/INR		0.99 ± 0.04	1.00 ± 0.04	0.593
APTT ratio		0.95 ± 0.07	0.92 ± 0.07	0.549
Blood urea nitrogen (mg/dL)		19.2 ± 7.7	9.0 ± 4.2	0.473
Creatinine (mg/dL)		0.89 ± 0.16	0.97 ± 0.31	0.343
AST (U/L)		34.0 ± 9.5	27.3 ± 11.4	0.306
Sodium (mmol/L)		137.2 ± 3.5	138.9 ± 2.3	0.463
Potassium (mmol/L)		3.9 ± 0.9	3.8 ± 0. 5	0.406
Smoking status				0.247
Non-smoker	31 (62%)	14 (53.8%)	17 (70.8%)	
History of smoking	3 (6%)	1 (3.8%)	2 (8.3%)	
Current smoker	16 (32%)	11 (42.3%)	5 (20.8%)	
Injury site				0.425
Left	25 (50%)	11 (42.3%)	14 (58.3%)	
Right	21 (42%)	12 (46.2%)	9 (37.5%)	
Bilateral	4 (8%)	3 (11.5%)	1 (4.2%)	
Number of ribs fractured	3.94 ± 2.05	4.08 ± 1.94	3.79 ± 2.21	0.341
< 3	11 (22%)	5 (19.2%)	6 (25%)	0.623
≥ 3	39 (78%)	21 (80.8%)	18 (75%)	0.623
Trauma mechanism				0.243
Car accident	6 (12%)	4 (15.4%)	2 (8.3%)	
Motorcycle accident	25 (50%)	10 (38.5%)	15 (62.5%)	
Pedestrian	2 (4%)	0	2 (8.3%)	
Fall < 3 m	12 (24%)	8 (30.8%)	4 (16.7%)	
Fall > 3 m	2 (4%)	2 (7.7%)	0	
Work accident	2 (4%)	1 (3.8%)	1 (4.2%)	
Fighting	1 (2%)	1 (3.8%)	0	
Associated injuries				
Fracture of extremity	4 (8%)	2 (7.7%)	2 (8.3%)	0.933
Head	1 (2%)	1 (3.8%)	0	0.332
Liver laceration	1 (2%)	1 (3.8%)	0	0.332
Scapular fracture	5 (10%)	2 (7.7%)	3 (12.5%)	0.571
Clavicle fracture	8 (16%)	2 (7.7%)	6 (25%)	0.095
Diabetes mellitus	12 (24%)	5 (19.2%)	7 (29.2%)	0.411
Hypertension	17 (34%)	7 (26.9%)	10 (41.7%)	0.272
Heart disease	5 (10%)	2 (7.7%)	3 (12.5%)	0.571
Kidney disease	3 (6%)	2 (7.7%)	1 (4.2%)	0.600
Anti-platelet/coagulant use before trauma	5 (10%)	2 (7.7%)	3 (12.5%)	0.571

*APTT* activated partial thromboplastin time, *AST* aspartate aminotransferase, *BMI* body mass index, *INR* international normalized ratio, *ISS* Injury Severity Score

### Pulmonary function test

There were no statistically significant between-group differences for %FVC (*p* = .371) or %FEV_1_ (*p* = .717) in the pre-test. In the control group, the pre-test %FVC was 70.44 ± 16.99% and the post-test %FVC was 65.58 ± 16.36%, representing a 4.86 ± 10.92% decrease. In the study group, the pre-test %FVC was 59.05 ± 15.34% and the post-test %FVC was 77.72% ± 13.28, representing an 18.65 ± 17.77% improvement (*p* < 0.001) (Table 2). There was no change in %FEV1 (−5.28 ± 11.95%) between the pre-test and post-test in the control group and a significant (19.49 ± 17.50%) improvement in the study group between the pre-test and post-test (*p* < 0.001).

**Table 2:** ANCOVA of pulmonary function test

Covariant	Control group (*n* = 26)	Study group (*n* = 24)	*p* value
%FEV_1_ pre-test	71.96 ± 16.33	60.48 ± 16.65	.717
%FEV_1_ post-test	66.66 ± 16.97	79.97 ± 13.04	.003*
%FEV_1_ difference	−5.28 ± 11.95	19.49 ± 17.50	< .001*
%FVC pre-test	70.44 ± 16.99	59.05 ± 15.34	.371
%FVC post-test	65.58 ± 16.36	77.72 ± 13.28	.006*
%FVC difference	−4.85 ± 10.92	18.65 ± 17.77	< .001*

*ANCOVA* analysis of covariance, *FEV*_*1*_ forced expiratory value in 1 s, *FVC* forced vital capacity

*p < 0.05

### Pulmonary complications

Altogether, 28 patients developed pulmonary complications, including 21 patients (75%) in the control group and 7 patients (25%) in the study group (*p* < 0.001). The most common complication was hemothorax (25 patients, 89.2%), including 18 patients (72%) in the control group. Five of these required a tube thoracostomy. In comparison, seven patients (28%) in the study group developed hemothorax (*p* = 0.005) (Table 3). One patient in the control group developed pneumothorax, but none in the study group, and two patients developed atelectasis. There were no cases of pneumonia in either group.

**Table 3:** Pulmonary complications

	Whole cohort	Control	Study	*t*-test	*p* value
	*N* = 50	*n* = 26	*n* = 24		
Pulmonary complications				11.538	.001*
No	22 (44%)	5 (19.2%)	17 (70.8%)		
Yes	28 (56%)	21 (80.7%)	7 (29.2%)		
Pneumothorax				0.942	.332
No	49 (98%)	25 (96.2%)	24 (100%)		
Yes	1 (2%)	1 (3.8%)	0		
Hemothorax				8.013	.005*
No	25 (50%)	8 (30.8%)	17 (70.8%)		
Yes	25 (50%)	18 (69.2%)	7 (29.2%)		
Pneumonia					
No	50 (100%)	26 (100%)	24 (100%)		
Yes	0	0	0		
Atelectasis				1.923	.166
No	48 (96%)	24 (92.3%)	24 (100%)		
Yes	2 (4%)	2 (7.7%)	0		
Tube thoracostomy	5 (10%)	5 (19.2%)	0	5.128	.051

*p < 0.05

### Length of hospitalization and pain score

The mean length of hospitalization was 9.98 ± 3.93 days, and there was no significant difference between the groups (Table 4). The mean score on the NRS for chest pain was 4.1 points and pain severity decreased between the 1st and 5th days of admission. The analgesic protocol achieved similar results in both groups.

**Table 4:** Length of stay and numeric rating scale of pain

	Whole cohort *N* = 50	Control *n* = 26	Study *n* = 24	χ^2^ / *t*	*p* value
Length of stay (days)	9.98 ± 3.93	9.92 ± 4.10	10.04 ± 3.82	0.313	.578
NRS					
1st day	4.34 ± 1.41	4.23 ± 1.45	4.46 ± 1.38	0.567	.361
2nd day	3.26 ± 1.38	3.38 ± 1.70	3.13 ± 0.95	0.660	.663
5th day	2.87 ± 1.33	3.00 ± 1.48	2.73 ± 1.17	0.686	.634

*NRS* numeric rating scale

## Discussion

Our results prove that IS use in patients with traumatic rib fractures can reduce pulmonary complications, including atelectasis and hemothorax, and further interventions. Moreover, IS use by patients with a traumatic rib fracture can improve their pulmonary function, as assessed by PFT, especially %FVC and %FEV_1_. Using an IS device did not prolong the length of hospitalization nor increase the severity of chest pain.

### Population of chest trauma

Our study population was predominantly male (68%). A study by Byun and Kim [27] conducted between 2002 and 2008 on 418 rib fracture patients also had more male patients (327 [78%] males and 91 [22%] females). Shulzhenko et al. [28] reviewed a database of 67,659 patients aged ≥65 years with traumatic rib fractures. There were 36,966 (54.6%) male patients and 30,715 (45.4%) female patients. Ekpe and Eyo [29] reported that 84.4% of patients with traumatic rib fractures were male (54 of 64 patients). In 2017, accidents were the sixth leading cause of death in Taiwan. There were 6965 fatalities, and 73% of the deaths were male [30]. Our study is consistent with these studies and proves that the high predominance of males in trauma studies may be because males often occupy high-risk jobs, such as in the transportation industry, and are more likely to ride motorcycles.

### Severity of chest trauma

The ISS standardizes the severity of traumatic injury, and a higher score may indicate higher mortality and morbidity. The lowest ISS in our study was 2 and the highest was 14, with a mean of 8.16 ± 3.17. Byun and Kim [27] found a mean ISS of 15.27 in 418 patients with rib fractures, with mortality and pneumonia rates that correlated with ISS (*p* < 0.001). In the database of 67,659 patients aged ≥65 years with traumatic rib fractures studied by Shulzhenko et al. [28], 24,128 people (35.6%) had an ISS of 1–9 in, 19,388 people (28.6%) an ISS of 10–16, and 22,825 people (33.7%) an ISS of 17–75. Butts et al. [31] reported the ISSs for 99 patients with rib fractures. The lowest score was 9 and the highest was 14, with a mean of 10. In our patients, the ISSs were much lower due to our exclusion criteria (ISS > 16), and we excluded post-trauma complications, such as pneumonia, which may be due to other types of severe trauma. Patient comorbidities, such as hyperglycemia and cardiovascular disease, can affect the post-trauma complication rate and mortality, and in particular, underlying pulmonary disease can increase the pneumonia rate after rib fracture [32]. We excluded patients who had chronic obstructive airway disease, asthma, or other pulmonary diseases at the beginning of the study.

### Beneficial effect of IS on surgical patients

The benefit of using an IS is that it forces the patient to take long deep breaths and hold them for seconds, thereby reducing breathing effort, decreasing the activity of accessory muscles, promoting diaphragmatic breathing, opening up the alveoli, improving tidal volume and basal ventilation, and consequently, encouraging the cleaning out of secretions. The preventive effect of IS on postoperative pulmonary complications has been proved by many studies. Renault et al. [33] randomly assigned 36 patients who underwent coronary artery bypass surgery into two groups: a deep breathing exercise group (*n* = 18) and an IS group (*n* = 18). Although there was no significant difference in FVC or FEV_1_ between the two groups, there were improvements in pulmonary function in the IS group. Alaparthi et al. [34] randomly assigned 260 patients who had undergone laparoscopic abdominal surgery into four groups, including a diaphragmatic breathing exercise group, a volume-flow IS group, and a control group. All patients had improved FVC and FEV_1_ on the second day after surgery, but the improvements in the flow IS group were more significant. Rollin et al. [35] carried out a retrospective study with 84 patients who had undergone laparoscopic donor nephrectomy. Five patients in the general care group developed pneumonia after surgery, but there were no cases of pneumonia in the IS group. Koo and Hwang [36] assigned 63 patients who had undergone upper abdominal surgery into a control group (*n* = 32) and an IS group (*n* = 32). Five patients (16.1%) in the control group developed pulmonary complications compared to none in the IS group (*p* = 0.18).

### Beneficial effects of IS on patients with chest trauma

The other beneficial effect of IS is on O_2_ saturation of post-traumatic rib fracture patients. Moon et al. [37] investigated 25 patients with rib fractures who used an IS after their admission to hospital. Their PFT results and O_2_ saturation levels improved day by day. In our study, 28 patients (56%) developed pulmonary complications after admission, 21 (75%) of whom were in the control group (*p* < 0.001). There were no cases of atelectasis in the IS group but two patients in the control group developed atelectasis (*p* = 0.166). There were no cases of pneumonia in our cohort. A total of 25 patients developed delayed hemothorax, 18 (72%) of whom were in the control group (*p* = 0.005). Five of these patients underwent a tube thoracostomy. A prospective study of minor blunt chest trauma by Misthos et al. found that delayed hemothorax was most frequently diagnosed in patients on the seventh day after blunt rib fracture, which is the main cause of hemothorax [38]. Whether IS can help to restore the alignment of fractured ribs and decrease cases of delayed hemothorax, as seen in our results, needs further study.

Despite the small sample size of our study, the results of this prospective randomized study prove that post-traumatic IS can effectively reduce post-traumatic pulmonary complications, including atelectasis, delayed hemothorax, and the need for a tube thoracotomy, in patients with traumatic rib fractures. Currently, the evidence-based therapeutic interventions for a rib fracture include only multimodal pain management, pulmonary hygiene, and operative stabilization [39]. The importance of pulmonary hygiene, the most commonly written order for patients with rib fractures, is a belief that is deeply held by practitioners. However, there is little evidence to show that this intervention for patients with rib fractures improves outcomes [40]. Our results provide direct evidence for the therapeutic effects of IS, besides the improvement in oxygen saturation, in preventing post-traumatic pulmonary complications in patients with rib fractures.

### Limitations

This study had several limitations. First, our relatively small sample size may not be representative of the population of rib fracture patients in Taiwan. We included only patients who were admitted to hospital and it is likely that patients who are not admitted also suffer from delayed pulmonary complications. This may limit the generalizability of our results to wider populations. In future, we may consider extending the inclusion criteria to encompass outpatients and patients attending an emergency department. Second, we performed pulmonary function testing only within 1 week of trauma, so the outcomes reported are relatively short term. Further studies on long-term outcomes and on training on IS compliance after discharge will help to further our knowledge of the benefits of IS. Third, our interventional procedures are challenging to blind, but the effort would be worthwhile by avoiding bias. However, blinding was reported to be successful in only a few interventional studies (13/63; 21%) [41].

## Conclusion

In conclusion, IS use reduced pulmonary complications, including atelectasis and hemothorax, and further interventions, such as a tube thoracostomy, in patients with rib fractures. Furthermore, the PFT results in patients who used an IS showed significant improvements in %FVC and %FEV_1_. The IS device does not extend the length of hospitalization or increase the severity of chest pain. These devices are easy to use and they have clinical benefits for patients with rib fractures without harmful effects.

## Data Availability

All data generated and analysed during this study are included in this published article.

## Abbreviations

ANCOVA: Analysis of covariance
BMI: Body mass index
CXR: Chest X-ray
FEV_1_: Forced expiratory volume in 1 s
FVC: Forced vital capacity
IS: Incentive spirometer
ISS: Injury Severity Score
NRS: Numeric rating scale
PFT: Pulmonary function test
